# Dissociation between liver fat content and fasting metabolic markers of selective hepatic insulin resistance in humans

**DOI:** 10.1093/ejendo/lvae123

**Published:** 2024-10-01

**Authors:** Felix A Westcott, Shilpa R Nagarajan, Sion A Parry, Dragana Savic, Charlotte J Green, Thomas Marjot, Elspeth Johnson, Thomas Cornfield, Ferenc E Mózes, Paige O’Rourke, Jessica Mendall, David Dearlove, Barbara Fielding, Kieran Smith, Jeremy W Tomlinson, Leanne Hodson

**Affiliations:** Oxford Centre for Diabetes, Endocrinology and Metabolism, University of Oxford, Oxford, OX3 7LE, United Kingdom; Oxford Centre for Diabetes, Endocrinology and Metabolism, University of Oxford, Oxford, OX3 7LE, United Kingdom; Oxford Centre for Diabetes, Endocrinology and Metabolism, University of Oxford, Oxford, OX3 7LE, United Kingdom; Aston Medical School, Aston University, Birmingham, B4 7ET, United Kingdom; Oxford Centre for Diabetes, Endocrinology and Metabolism, University of Oxford, Oxford, OX3 7LE, United Kingdom; Oxford Centre for Clinical Magnetic Resonance Research, University of Oxford, Oxford, OX3 9DU, United Kingdom; Oxford Centre for Diabetes, Endocrinology and Metabolism, University of Oxford, Oxford, OX3 7LE, United Kingdom; Oxford Centre for Diabetes, Endocrinology and Metabolism, University of Oxford, Oxford, OX3 7LE, United Kingdom; Oxford Centre for Diabetes, Endocrinology and Metabolism, University of Oxford, Oxford, OX3 7LE, United Kingdom; Oxford Centre for Diabetes, Endocrinology and Metabolism, University of Oxford, Oxford, OX3 7LE, United Kingdom; Oxford Centre for Clinical Magnetic Resonance Research, University of Oxford, Oxford, OX3 9DU, United Kingdom; Oxford Centre for Diabetes, Endocrinology and Metabolism, University of Oxford, Oxford, OX3 7LE, United Kingdom; Oxford Centre for Diabetes, Endocrinology and Metabolism, University of Oxford, Oxford, OX3 7LE, United Kingdom; Oxford Centre for Diabetes, Endocrinology and Metabolism, University of Oxford, Oxford, OX3 7LE, United Kingdom; Oxford Centre for Diabetes, Endocrinology and Metabolism, University of Oxford, Oxford, OX3 7LE, United Kingdom; Faculty of Health and Medical Sciences, University of Surrey, Guildford, GU2 7XH, United Kingdom; Oxford Centre for Diabetes, Endocrinology and Metabolism, University of Oxford, Oxford, OX3 7LE, United Kingdom; Oxford Centre for Diabetes, Endocrinology and Metabolism, University of Oxford, Oxford, OX3 7LE, United Kingdom; OCDEM, National Institute for Health Research Oxford Biomedical Research Centre, Oxford University Hospital Trusts, Oxford, OX3 7LE, United Kingdom; Oxford Centre for Diabetes, Endocrinology and Metabolism, University of Oxford, Oxford, OX3 7LE, United Kingdom; OCDEM, National Institute for Health Research Oxford Biomedical Research Centre, Oxford University Hospital Trusts, Oxford, OX3 7LE, United Kingdom

**Keywords:** insulin resistance, gluconeogenesis, *de novo* lipogenesis, liver fat, hyperinsulinemia

## Abstract

**Objective:**

Fasting hyperglycemia and hypertriglyceridemia are characteristic of insulin resistance (IR) and rodent work has suggested this may be due to selective hepatic IR, defined by increased hepatic gluconeogenesis and *de novo* lipogenesis (DNL), but this has not been shown in humans.

**Design:**

Cross-sectional study in men and women across a range of adiposity.

**Methods:**

Medication-free participants (*n* = 177) were classified as normoinsulinemic (NI) or hyperinsulinemic (HI) and as having low (LF) or high (HF) liver fat content measured by magnetic resonance spectroscopy. Fractional gluconeogenesis (frGNG) and hepatic DNL were measured using stable isotope tracer methodology following an overnight fast.

**Results:**

Although HI and HF groups had higher fasting plasma glucose and triglyceride concentrations when compared to NI and LF groups respectively, there was no difference in frGNG. However, HF participants tended to have lower frGNG than LF participants. HI participants had higher DNL compared to NI participants but there was no difference observed between liver fat groups.

**Conclusions:**

Taken together, we found no metabolic signature of selective hepatic IR in fasting humans. DNL may contribute to hypertriglyceridemia in individuals with HI but not those with HF. Glycogenolysis and systemic glucose clearance may have a larger contribution to fasting hyperglycemia than gluconeogenesis, especially in those with HF, and these pathways should be considered for therapeutic targeting.

## Graphical Abstract

**Graphical Abstract:**
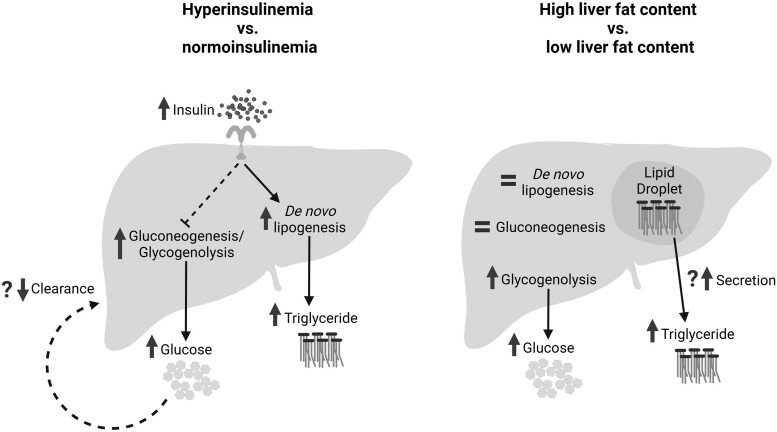
Compared to participants with normoinsulinemia, those with hyperinsulinemia had higher *de novo* lipogenesis which may explain their elevated plasma triglyceride concentration. An increase in both gluconeogenesis and glycogenolysis (as well as a possible decrease in glucose clearance) may drive the higher plasma glucose levels in those with hyperinsulinemia compared to normoinsulinemia. Participants with high liver fat content had no change in either *de novo* lipogenesis or gluconeogenesis compared to those with low liver fat content, and the elevated plasma glucose and triglyceride concentrations may be driven by increased glycogenolysis and triglyceride secretion from lipid droplets, respectively.

**Significance**

Selective hepatic insulin resistance has the metabolic signature of increased hepatic gluconeogenesis and *de novo* lipogenesis and has been suggested to be the underlying mechanism for hyperglycemia and hypertriglyceridemia but evidence in humans is lacking. By studying men and women, across a range of liver fat content and fasting plasma insulin concentrations, we did not observe a clear metabolic signature of selective hepatic insulin resistance as hepatic gluconeogenesis and *de novo* lipogenesis together did not discriminate participants classified as hyper- compared to normo-insulinemic or with high compared to low liver fat content. Therefore, glycogenolysis and systemic glucose clearance may play a larger role, than gluconeogenesis, in contributing to fasting hyperglycemia than previously thought and should be considered for therapeutic targeting.

## Introduction

Insulin resistance (IR) is strongly associated with obesity and often coexists with type 2 diabetes (T2D) and metabolic dysfunction-associated steatotic liver disease (MASLD) and is characterized by elevated circulating levels of insulin, glucose, and triglycerides. Insulin orchestrates the switching of hepatic metabolism from energy release in the fasting state to energy storage in the postprandial state; it is thought that aberrant hepatic insulin signaling may dysregulate these processes in IR.^1^ Two main pathways commonly associated with IR are: (1) gluconeogenesis (GNG), the synthesis of new glucose from gluconeogenic precursors, and (2) *de novo* lipogenesis (DNL), the synthesis of new fatty acids (FAs) from non-lipid precursors. Insulin indirectly regulates hepatic GNG through the flux of adipose- (glycerol) and musculoskeletal-derived (alanine, lactate, glutamine) GNG precursors, whereas insulin has a direct effect on hepatic DNL and glycogen breakdown (glycogenolysis, GLY). In insulin-resistant states, insulin may lose its inhibitory control on hepatic glucose production but retain its direct stimulatory effect on DNL.^2,3^ This divergence in insulin sensitivity has been referred to as “selective hepatic insulin resistance” and has been suggested to drive the accumulation of liver fat, along with the increased circulating levels of glucose, insulin, and triglycerides associated with IR.^3-5^

The distinction between selective and total hepatic IR derives from murine studies with liver-specific insulin receptor knockout that displayed both impaired suppression of glucose production and impaired stimulation of DNL. This is consistent with the presence of hyperglycemia, hyperinsulinemia, and lack of hepatic steatosis observed in humans with mutations in the insulin receptor.^6,7^ While several studies performed in rodents and canines have focused on dissecting the molecular mechanisms of selective hepatic IR,^8-10^ its existence in humans has yet to be substantiated.

In humans, elevated plasma insulin concentrations, DNL, and liver fat accumulation often coexist, although it is not clear which comes first. Compared to hepatic DNL, GNG and GLY are rarely measured; thus, determining their contributions in response to IR is challenging. Therefore, the aim of this study was to investigate whether the proposed metabolic markers of selective hepatic IR (ie, increased hepatic GNG and DNL) are associated with hyperinsulinemia or liver fat content in humans in the fasted state.

## Materials and methods

### Participants

Participants (*n* = 177, 101 female and 76 male) were recruited from the Oxford BioBank (www.oxfordbiobank.org.uk),^11^ by advertisement and from hepatology clinics at the Oxford University Hospital Trust, Oxford. Volunteers were not taking medication known to affect lipid or glucose metabolism, did not smoke, and did not consume alcohol above recommended limits. Eighty-six individuals (49%) were considered hyperinsulinemic (HI) with fasting plasma insulin concentrations greater than the 75th centile (11.2 U/mL) of the Oxford BioBank,^12,13^ and 91 were considered normoinsulinemic (NI). When split by liver fat, 60 participants (34%) were considered to have high liver fat (HF) with a liver fat content higher than the 95th centile (5.56% liver fat) of the Dallas Heart Study,^14^ and 117 participants were considered to have low liver fat (LF). The participants were then further grouped as normoinsulinemic with low liver fat content (NILF), normoinsulinemic with high liver fat content (NIHF), hyperinsulinemic with low liver fat content (HILF), or hyperinsulinemic with high liver fat content (HIHF). Two participants were considered to have type 2 diabetes (T2D) based on the American Diabetes Association’s fasting blood glucose concentrations cutoff of >7 mmol/L.^15^ The exclusion of these participants did not change the results on reanalysis. As these individuals did not have a diagnosis of T2D at the time of recruitment, they were included in the final dataset. The studies were approved by the Oxfordshire, Portsmouth, North of Scotland, North West–Lancaster, North East–Tyne and Wear, and South Clinical Research Ethics Committees and all research complied with the Declaration of Helsinki. All participants gave written informed consent. Some, but not all, of the data reported in this work constitute a reanalysis of previously published studies,^13,16-20^ and only data from the fasting time point of the pre-intervention/baseline study day has been included in this study.

### Study protocol

All participants were asked to avoid alcohol and strenuous exercise for 24 hours prior to their study day. The evening prior to the study day, participants consumed ^2^H_2_O (3 g/kg body water, split into two doses to minimize dizziness) for the measurement of fasting hepatic GNG and DNL. Participants attended the clinical research unit the next morning after an overnight fast. Body composition and body fat percentage were measured using dual-energy X-ray absorptiometry, and a fasting venous blood sample was collected from an antecubital vein. Liver fat content was measured within 1 week of the study day using magnetic resonance spectroscopy.^13^

### Analytical methods

Whole blood was collected into heparinized syringes (Sarstedt, Leicester, United Kingdom) and centrifuged at 4 °C to isolate plasma. Concentrations of plasma glucose, glycerol, non-esterified FA (NEFA), total and high-density lipoprotein (HDL) cholesterol, triglyceride (TG), and 3-hydroxybutyrate (3-OHB) were analyzed on a semi-automatic analyzer (ILab 600/650 clinical chemistry; Warrington, United Kingdom). Plasma insulin and glucagon levels were determined through enzyme-linked immunosorbent assays (Mercodia, Uppsala, Sweden). Fasting plasma alanine concentrations (*n* = 130) were measured using a nuclear magnetic resonance (NMR) metabolomics platform (Nightingale Health, Helsinki, Finland). Very-low-density lipoprotein (VLDL) fractions (Svedberg flotation 20-400) were isolated using density gradient ultracentrifugation.^13^

### Measurement of DNL

Triglyceride was extracted from VLDL fractions and FA methyl esters were prepared. FA compositions (µmol/100 µmol total FA) were determined using gas chromatography (GC), which were subsequently used to calculate palmitate (16:0) concentrations.^12^ Tracer enrichments in extracted VLDL-TG fractions were determined by GC–mass spectrometry (MS).^12^ Deuterium incorporation from ^2^H_2_O in plasma water (Finnigan GasBench II; Thermo Fisher Scientific, Paisley, United Kingdom) into VLDL-TG palmitate was determined using GC–MS monitoring ions with mass-to-charge ratios (m/z) of 270 (M + 0) and 271 (M + 1).^21^  *De novo* lipogenesis was calculated as the percentage contribution of newly synthesized 16:0 to VLDL-TG (fractional DNL, frDNL) which could then be multiplied by the plasma concentration of VLDL-TG to give an absolute concentration of newly synthesized 16:0 in VLDL-TG.

### Measurement of GNG and GLY

Glucose was extracted from participant plasma based on a previously described method.^22^ Briefly, plasma was deproteinized with ethanol and methylhydroxylamine hydrochloride in pyridine (2% w/v) was added to the dried supernatant. Samples were then heated and cooled and N, O Bis(trimethylsilyl)trifluoroacetamide (BSTFA) + 1% Trimethylchlorosilane (TCMS) added, before being reheated, cooled, and dried. Samples were reconstituted in decane, and tracer enrichment was analyzed by GC–MS monitoring for ions with a mass-to-charge ratio (m/z) of 319 (M + 0) and 320 (M + 1). ^2^H_2_O enrichment in plasma water was measured, and the contribution of GNG to plasma glucose concentration (fractional GNG, frGNG) was calculated using the “average” method.^23^ This method was adapted to reflect the number of hydrogens on our derivative^22^ and was validated by a strong positive correlation with the original method (Figure S1). Fractional GLY was determined as the inverse of frGNG.^24^ As endogenous glucose production was not measured, absolute rates of GNG and GLY could not be calculated. However, to account for differences in pool sizes, the plasma glucose concentration derived from GNG and GLY was calculated by multiplying plasma glucose concentration by fractional GNG and GLY, respectively.

### Calculations and statistical analysis

The homeostatic model assessment for insulin resistance (HOMA-IR) was calculated based on fasting plasma glucose and insulin concentrations.^25^ The glucagon–alanine index, a marker of hepatic glucagon signaling, was calculated by multiplying fasting plasma glucagon and alanine concentrations.^26^ All statistical analysis was performed in R (version 4.2.2 for Windows, RStudio, United States). For comparisons between groups, chi-squared tests were used for categorical data, and continuous data was tested for normality followed by independent *t*-tests or non-parametric equivalent using the Benjamini–Hochberg correction for multiple comparisons if required. Pearson's correlation coefficient is presented for normally distributed variables with a linear relationship; otherwise, Spearman's rank correlation coefficient is used. For multivariate analysis, independent variables were entered if they exhibited a significant univariate association, and the model was then refined through backward elimination. Data in box plots are presented as median with quartiles and scatterplots fitted with linear regression line and 95% confidence interval. Statistical significance was set at *P* < .05.

## Results

### Participant characteristics

Age and sex did not differ between the NI and HI groups, but those defined as HI had a higher (*P* < .001) body mass index (BMI), fat mass, liver fat content, and glucagon–alanine index (Table 1). Fasting plasma glucose, glucagon, and TG concentrations were higher (*P* < .001) in the HI compared to the NI group (Table 1). When comparing individuals defined as either LF or HF, we found no difference in age between the groups, but there were a greater proportion of men in the HF compared to the LF group (60 vs 34% respectively, *P* < .01). Those in the HF group had a higher (*P* < .001) BMI, fat mass, HOMA-IR, and glucagon–alanine index, along with greater fasting plasma glucose, insulin, glucagon, and TG concentrations compared to the LF group (Table 2).

**Table 1.** Participant characteristics defined by fasting plasma insulin.

	NI (≤11.2 U/mL) *n* = 91	HI (>11.2 U/mL) *n* = 86
Female/male	57/34	44/42
Age (y)	48 ± 1	47 ± 1
BMI (kg/m^2^)	25.6 (19.5-35.2)	29.5 (21.7-45.6)^a^
Fat mass (%)	33 ± 1	37 ± 1^a^
Lean mass (%)	67 ± 1	63 ± 1^a^
IHTG (%)	2 (0-24)	6 (0-38)^a^
HOMA-IR	1.9 (0.2-2.8)	3.6 (2.3-23.5)^a^
Glucagon–alanine index^b^	1.3 (0.0-5.6)	3.4 (0.1-10.9)^a^
*Fasting plasma biochemistry*		
Glucose (mmol/L)	5.0 (3.4-6.2)	5.5 (4.5-11.2)^a^
Insulin (U/mL)	9 (1-11)	15 (11-72)^a^
Glucagon (pmol/L)	5 (0-29)	9 (0-46)^a^
NEFA (µmol/L)	513 (93-1093)	536 (172-1252)
TG (mmol/L)	0.9 (0.3-3.9)	1.4 (0.3-4.5)^a^
3OHB (µmol/L)	53 (15-580)	57 (14-560)
Total chol (mmol/L)	5.1 ± 0.1	5.0 ± 0.1
HDL chol (mmol/L)	1.4 (0.4-2.6)	1.1 (0.3-2.2)^a^
Non-HDL chol (mmol/L)	3.6 (1.9-6.7)	3.8 (2.2-6.4)
Lactate (mmol/L)	0.9 (0.5-2.4)	1.0 (0.6-1.8)^c^
Alanine (mmol/L)^b^	0.31 ± 0.01	0.31 ± 0.01
Glycerol (µmol/L)	47 (11-172)	46 (15-164)

Data presented as mean ± sem or median (min-max). Abbreviations: BMI, body mass index; IHTG, intrahepatic triglyceride; HOMA IR, homeostatic model assessment for insulin resistance; NEFA, non-esterified fatty acid; TG, triglyceride; 3OHB, 3-hydroxybutyrate; HDL, high-density lipoprotein; Chol, cholesterol. ^a^*P* < 0.001 and ^c^*P*< 0.05 normoinsulinemic (NI) vs hyperinsulinemic (HI) groups. ^b^Measured in 117 participants.

**Table 2.** Participant characteristics defined by liver fat content.

	LF (≤5.56%) *n* = 117	HF (>5.56%) *n* = 60
Female/male	77/40	24/36^a^
Age (y)	47 ± 1	48 ± 1
BMI (kg/m^2^)	25.6 (19.5-35.6)	30.5 (23.5-45.6)^b^
Fat mass (%)	34 ± 1	38 ± 1^b^
Lean mass (%)	66 ± 1	62 ± 1^b^
IHTG (%)	1 (0-5)	12 (6-38)^b^
HOMA-IR	2.2 (0.2-4.9)	4.3 (1.0-23.5)^b^
Glucagon–alanine index^c^	1.4 (0.0-5.0)	4.1 (0.5-10.9)^b^
*Fasting plasma biochemistry*		
Glucose (mmol/L)	5.1 (3.4-6.6)	5.5 (3.9-11.2)^b^
Insulin (U/mL)	10 (1-21)	17 (4-72)^b^
Glucagon (pmol/L)	5 (0-35)	13 (1-46)^b^
NEFA (µmol/L)	497 (93-1093)	562 (218-1252)
TG (mmol/L)	0.9 (0.3-4.1)	1.7 (0.3-4.5)^b^
3OHB (µmol/L)	54 (15-580)	55 (14-301)
Total chol (mmol/L)	5.0 ± 0.1	5.0 ± 0.1
HDL chol (mmol/L)	1.4 (0.4-2.6)	1.1 (0.3-1.8)^b^
Non-HDL chol (mmol/L)	3.6 (1.9-6.4)	3.9 (2.2-6.7)
Lactate (mmol/L)	0.9 (0.5-2.4)	1.0 (0.6-1.8)^a^
Alanine (mmol/L)^c^	0.30 ± 0.01	0.32 ± 0.01
Glycerol (µmol/L)	45 (11-172)	48 (17-164)

Data presented as mean ± sem or median (min-max). Abbreviations: BMI, body mass index; IHTG, intrahepatic triglyceride; HOMA IR, homeostatic model assessment for insulin resistance; NEFA, non-esterified fatty acid; TG, triglyceride; 3OHB, 3-hydroxybutyrate; HDL, high-density lipoprotein; Chol, cholesterol. ^a^*P* < 0.01 and ^b^*P* < 0.001 low (LF) vs high (HF) liver fat groups. ^c^Measured in 117 participants.

### Effect of fasting plasma insulin and liver fat content on fasting GNG and GLY

**Figure 1.**
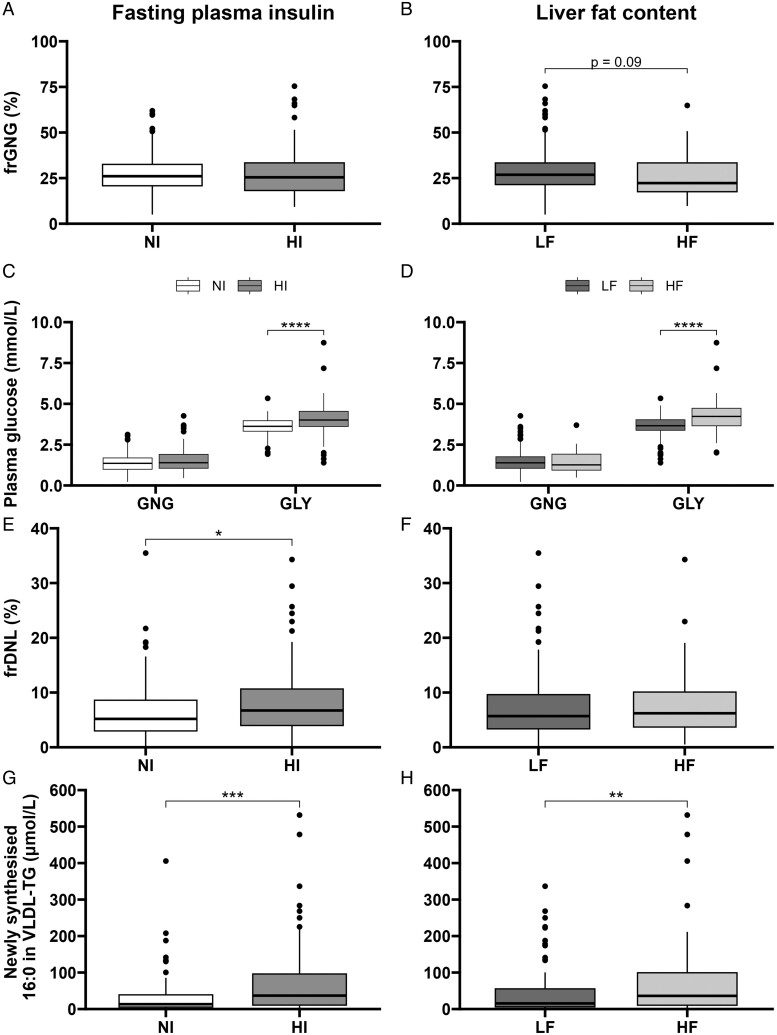
The effect of fasting plasma insulin and liver fat content on fasting GNG, GLY, and DNL. Participants (*n* = 177) were classified as normoinsulinemic or hyperinsulinemic (NI or HI) and as having low or high liver fat content (LF or HF). (A and B) Fractional gluconeogenesis (frGNG) was measured across the groups, and (C and D) the absolute contributions of gluconeogenesis (GNG) and glycogenolysis (GLY) to fasting plasma glucose concentration were calculated. (E and F) Fractional *de novo* lipogenesis across groups (frDNL) and (G and H) absolute contributions of newly synthesized 16:0 to very-low-density lipoprotein-triglyceride (VLDL-TG). *P < .05, **P < .01, ***P < .001, ****P < .0001, or *P*-value stated.

We found no difference in frGNG between HI and NI groups, but there was a trend (*P* = .09) toward a lower frGNG in the HF compared to the LF group (Figure 1A and B). The absolute concentration of glucose from GNG did not differ between the HI and NI groups nor the HF and LF groups (Figure 1C and D). The absolute concentration of glucose from GLY was higher in the HI and HF compared to NI and LF groups, respectively.

### Effect of fasting plasma insulin and liver fat content on fasting DNL

To examine the association of fasting DNL with hyperinsulinemia and liver fat content, we measured frDNL and found it to be higher in participants in the HI compared to the NI group (6.2 vs 5.2%, *P* = .034); however, there was no difference between the LF and HF groups (Figure 1E and F). The absolute concentration of DNL-derived FA to VLDL-TG was greater in participants in the HI and HF groups compared to the NI and LF groups, respectively (Figure 1G and H). Due to the significantly higher proportion of men in the HF compared to the LF group, all analyses were repeated on female and male participants separately. However, similar patterns were observed in both male and female participants suggesting observations were not sex-dependent (Figures S2 and S3).

### Further phenotypic grouping

**Figure 2.**
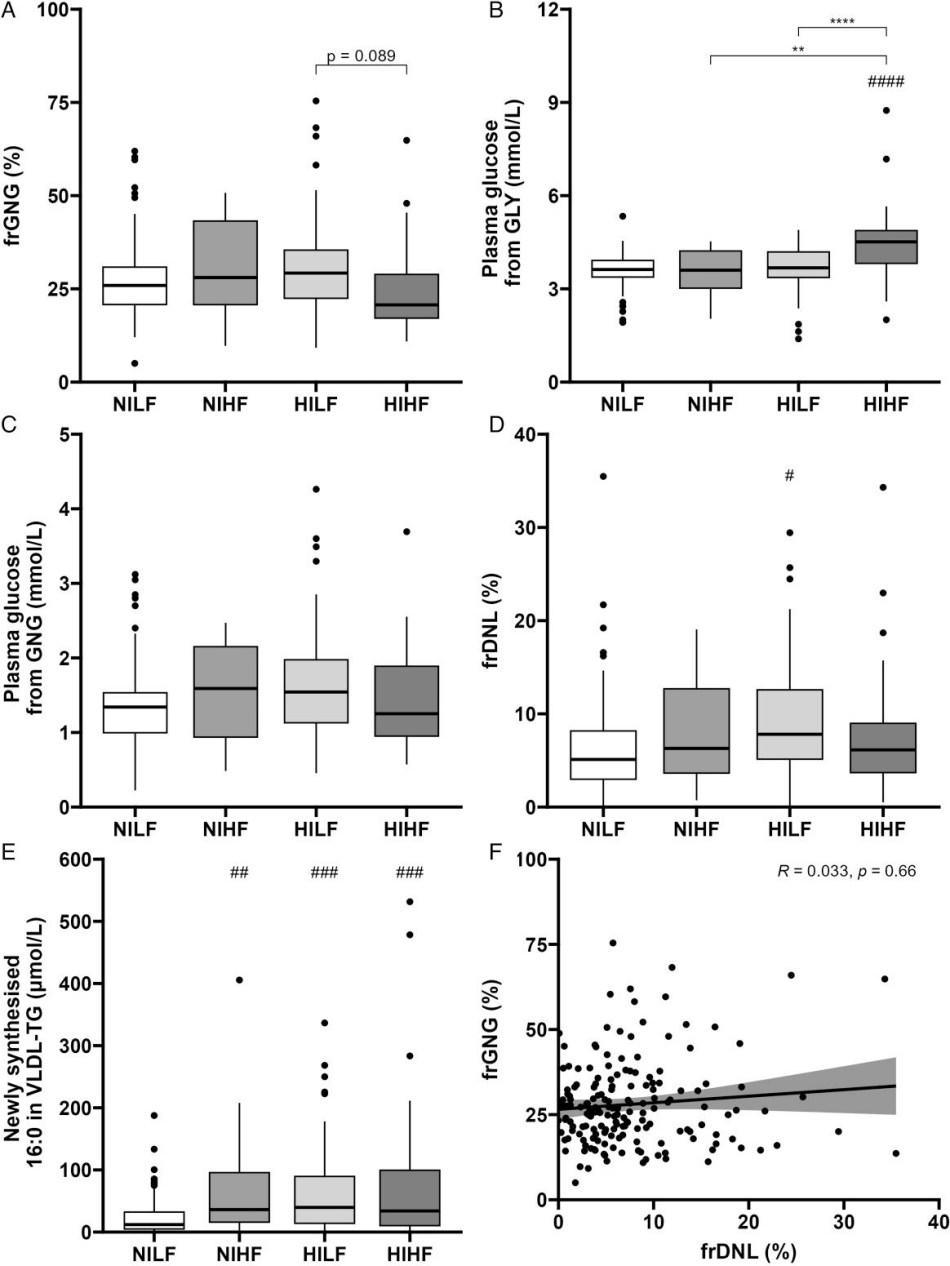
Participants were grouped as normoinsulinemic with low liver fat content (NILF), normoinsulinemic with high liver fat content (NIHF), hyperinsulinemic with low liver fat content (HILF), or hyperinsulinemic with high liver fat content (HIHF). (A) Fractional gluconeogenesis (frGNG) was measured, and the absolute contributions of (B) glycogenolysis (GLY) and (C) gluconeogenesis (GNG) to fasting plasma glucose concentration were calculated. (D) Fractional *de novo* lipogenesis (frDNL) across groups and (E) absolute contributions of newly synthesized 16:0 to very-low-density lipoprotein-triglyceride (VLDL-TG). (F) A correlation was performed across all participants between fractional gluconeogenesis (frGNG) and fractional *de novo* lipogenesis (frDNL). *P < .05, **P < .01, ***P < .001, and ****P < .0001 compared to either NILF or LGLD group and ^##^*P* < .01, ^###^*P* < .001, and ^####^*P* < .0001 for all other comparisons shown or *P*-value stated.

To explore the impact of both plasma insulin and liver fat content together, participants were characterized as NILF (*n* = 75), NIHF (*n* = 16), HILF (*n* = 42), or HIHF (*n* = 44). The NIHF and HIHF groups had a significantly greater proportion of male participants compared to the NILF group (Table 3). There was no difference in age between the groups, but participants in the NILF group had lower BMI compared to participants in the other groups. While those in the NILF group had higher HDL cholesterol but lower plasma glucagon, TG, and glucagon–alanine index compared to the other groups, those in the HIHF and HILF groups had greater fasting plasma glucose compared to the NILF group (Table 3). We found no significant differences in frGNG between the groups, although there was a trend (*P =* .089) for lower frGNG in HIHF compared to HILF (Figure 2A). There was no difference in the absolute concentration of glucose from GNG or GLY between the groups, although the HIHF group had a greater absolute concentration of glucose from GLY than all other groups (Figure 2B and C). Fractional *de novo* lipogenesis was higher in the HILF compared to the NILF group, with the latter having a lower absolute contribution of DNL to VLDL-TG compared to all other groups (Figure 2D and E). Similar findings were observed when analyzed by sex (Figures S4 and S5).

**Table 3.** Participant characteristics defined by phenotype.

	NILF *n* = 75	NIHF *n* = 16	HILF *n* = 42	HIHF *n* = 44
Female/male	54/21	3/13^a^	23/19	21/23^b^
Age (y)	48 ± 1	47 ± 2	46 ± 1	48 ± 1
BMI (kg/m^2^)	25.5 (19.5-31.3)	27.2 (24.2-35.2)^c^	27.3 (21.7-35.6)^c^	33.4 (23.5-45.6)^a^
Fat mass (%)	33 ± 1	33 ± 2	35 ± 1	40 ± 1^a^
Lean mass (%)	67 ± 1	67 ± 2	65 ± 1	60 ± 1^a^
IHTG (%)	1 (0-5)	7 (6-24)^a^	2 (0-5)	14 (6-38)^a^
HOMA-IR	1.9 (0.2-2.8)	2.0 (1.0-2.6)	3.1 (2.3-4.9)^a^	5.2 (2.9-23.5)^a^
Glucagon–alanine index^d^	1.2 (0.0-3.8)	2.8 (0.7-5.6)^b^	1.9 (0.1-5.0)^b^	4.4 (0.5-10.9)^a^
*Fasting plasma biochemistry*				
Glucose (mmol/L)	5.0 (3.4-6.2)	5.1 (3.9-5.7)	5.2 (4.5-6.6)^a^	5.7 (4.5-11.2)^a^
Insulin (U/mL)	9 (1-11)	9 (4-11)	13 (11-21)^a^	20 (13-72)^a^
Glucagon (pmol/L)	5 (0-29)	9 (1-27)^b^	7 (0-35)^b^	16 (1-46)^a^
NEFA (µmol/L)	532 (93-1093)	508 (237-873)	455 (172-874)	578 (218-1252)
TG (mmol/L)	0.8 (0.3-2.0)	1.6 (0.5-3.9)^a^	1.1 (0.3-4.1)^b^	1.7 (0.3-4.5)^a^
3OHB (µmol/L)	56 (15-580)	40 (17-273)	50 (22-560)	60 (14-301)
Total chol (mmol/L)	5.0 ± 0.1	5.3 ± 0.3	5.1 ± 0.1	4.9 ± 0.1
HDL chol (mmol/L)	1.5 (0.4-2.6)	1.1 (0.5-1.3)^a^	1.2 (0.6-2.2)^b^	1.0 (0.3-1.8)^a^
Non-HDL chol (mmol/L)	3.4 (1.9-6.0)	4.2 (2.2-6.7)^b^	3.7 (2.2-6.4)	3.8 (2.2-6.1)
Lactate (mmol/L)	0.8 (0.5-2.4)	1.0 (0.7-1.1)	0.9 (0.6-1.3)	1.1 (0.6-1.8)^b^
Alanine (mmol/L)^d^	0.30 ± 0.01	0.34 ± 0.02	0.31 ± 0.01	0.31 ± 0.01
Glycerol (µmol/L)	51 (11-172)	38 (23-114)	37 (15-114)	49 (17-164)

Data presented as mean ± sem or median (min-max). Abbreviations: BMI, body mass index; IHTG, intrahepatic triglyceride; HOMA IR, homeostatic model assessment for insulin resistance; NEFA, non-esterified fatty acid; TG, triglyceride; 3OHB, 3-hydroxybutyrate; HDL, high-density lipoprotein; Chol, cholesterol. ^a^*P* < 0.001; ^b^*P* < 0.01 and ^c^*P* < 0.05 compared to the NILF group. ^d^Measured in 117 participants.

### The associations between fasting GNG and DNL and multivariate analysis

We assessed the association between fasting frGNG and frDNL across all participants and found no correlation (Figure 2F). Multiple logistic regression was performed to determine which variables best predicted participant insulin group (HI or NI) and liver fat content group (HF or LF). The main predictors for plasma insulin were BMI (*β*= 0.179, *P* = .002), fasting plasma glucose (*β* = 1.71, *P* < .001), and glucagon (*β*= 0.0816, *P* = .018) concentrations (pseudo-R^2^ = 0.379), whereas the main predictors for liver fat content were glucagon–alanine index (*β* = 0.667, *P* = .003), HOMA-IR (*β* = 0.970, *P* = .002), and fasting plasma HDL cholesterol (*β*=−4.03, *P* = .003) and glycerol (*β* = 0.0524, *P* < .001) concentrations (pseudo-R^2^ = 0.766). Neither frGNG nor frDNL was a significant predictor in either model.

## Discussion

Individuals with IR, T2D, and MASLD have elevated fasting plasma glucose and TG concentrations compared to those without metabolic diseases.^27^ Findings from rodent models have suggested this may be due to selective resistance to insulin suppressing the GNG pathway but a preservation of insulin sensitivity in stimulating the DNL pathway.^3^ However, as most studies have focused on either the hepatic GNG or DNL pathway, it remains unclear if both pathways are concurrently upregulated in humans. Therefore, we investigated whether these signatures of selective hepatic IR were evident in a large cohort of participants across a range of fasting plasma insulin concentrations and liver fat content. Overall, we found no clear metabolic signature of increased fasting hepatic GNG and DNL that discriminated between individuals classified by insulinemia or liver fat content, nor did we observe an association between frGNG and frDNL. Thus, it is plausible these signatures may only be detected in a larger number of individuals with a more exacerbated phenotype (eg, severe obesity and liver fat content).

In-line with others,^28,29^ individuals classified with HI or HF had significantly higher plasma glucose concentrations compared to those classified as NI and LF, respectively. Although increased fasting glucose concentrations are often suggested to be driven by increased hepatic GNG in those with metabolic diseases,^29,30^ we found that the fractional and absolute contributions of GNG to plasma glucose are similar between HI and NI individuals. Others that utilized ^2^H_2_O to measure GNG and GLY also reported no difference in fasting frGNG between individuals with and without T2D despite notable differences in plasma glucose concentrations.^31-33^ In conjunction with our observations, this suggests GNG and GLY may contribute equally to the elevated fasting plasma glucose levels observed in individuals with IR. However, it has been suggested that the glucose produced from GLY may come from the simultaneous synthesis and breakdown of glycogen, which has been shown to be increased in patients with T2D.^33,34^ Alternatively, the elevation in fasting plasma glucose may come from decreased glucose clearance as observed in those with IR.^35,36^ The HI and HF groups in the current study had moderately raised plasma glucose concentrations, and it is likely that increased fasting frGNG may only be observed in those with severe hyperglycemia,^37,38^ suggesting GNG may have a larger contribution to fasting hyperglycemia in those with more severe IR.

The previous work reported no association between frGNG and liver fat content,^39^ and we found no difference in frGNG between the HF and LF groups. However, we did observe a tendency for lower frGNG in those with HF compared to LF, suggesting elevated fasting plasma glucose concentrations may be largely driven by GLY rather than GNG. Excess hepatocellular glycogen is common in MASLD patients and is associated with higher fasting plasma glucose concentrations.^40^ It is plausible that those with HF have higher hepatic glycogen stores and utilize this pool more for glucose production than LF individuals, contributing to a rise in plasma glucose concentration. The breakdown of hepatic glycogen is stimulated by circulating glucagon that may be elevated in those with HF. It has been shown that glucagon-mediated hepatic ureagenesis is impaired in people with hepatic steatosis that fosters a hyperglucagonemic response to normalize amino acid concentrations.^26^ Such hyperglucagonemia may drive an increase in hepatic glucose production primarily through GLY rather than GNG.^41^ In our analysis, there was a strong positive association between liver fat content and the glucagon–alanine index suggesting an impairment in glucagon signaling at the level of hepatic amino acid metabolism. Similarly, when participants were classified by both plasma insulin and liver fat content, HIHF individuals had a significantly higher contribution of GLY to fasting plasma glucose concentrations compared to the other respective groups while also tending to have lower frGNG than those in the HILF group. Overall, this suggests that HI may be associated with an equal increase in both GNG and GLY and/or a decrease in glucose clearance, whereas HF is associated with a specific increase in GLY only. It is therefore plausible that HF-induced disruptions in the liver–α-cell axis alter fasting glycemia by increasing GLY which is in-line with others who have reported a disruption in liver–α-cell axis associated with liver fat content.^42^

In the current study, fasting plasma TG concentrations were higher in HI and HF compared to NI and LF groups, consistent with the previous work.^13,28^ It has been suggested that increased plasma TG concentrations may be driven by hepatic DNL, which tends to be increased in obese and IR populations.^13,43,44^ We found frDNL was higher in those with HI compared to NI; however, it did not differ between the HF and LF groups, although absolute concentrations of DNL-derived FA to VLDL-TG were significantly higher in both HI and HF compared to NI and LF, respectively, which may in part be reflective of a larger VLDL-TG pool size. When participants were classified by plasma insulin and liver fat, only those in the HILF group had significantly higher frDNL compared to NILF participants. Hepatic DNL may, therefore, be driven by hyperinsulinemia, rather than liver fat content alone. We have previously reported higher frDNL in individuals with HI compared to NI, despite a large overlap in liver fat content between groups.^13^ Previous work has shown higher frDNL in obese compared to lean individuals who had similar liver fat content, but the hepatic insulin sensitivity index was lower in the obese group,^45^ supporting the concept that DNL is associated with insulin sensitivity rather than liver fat content. Moreover, based on previous observations,^46,47^ the FA composition of liver fat, rather than the total quantity, may have a greater effect on DNL.

Our study is not without limitations. Although fasting plasma glucose and insulin are frequently used as clinical biomarkers for IR, it would be of interest to measure the dynamic changes in GNG, GLY, and DNL following a provocation (eg, meal consumption or insulin/glucagon infusion). Similarly, we measured the contributions of GNG and GLY to fasting plasma glucose concentration, but additional measurement of endogenous glucose production to calculate the absolute rates of GNG and GLY may increase the sizes of the effects. Participants consumed ^2^H_2_O the evening prior to the study day, for the assessment of hepatic DNL, as reported by others.^48-50^ However, it has been suggested that a longer labeling period is required in individuals with liver fat accumulation, as the turnover of the pools is slow (t_½_ > 10 days).^51^ Thus, we may have underestimated the contribution of DNL-derived FA to VLDL-TG in individuals with HF and may explain why our fasting values in individuals with HF are lower than what has previously been reported when a longer period of ^2^H_2_O consumption has been utilized.^44^ The majority of the cohort studied were Caucasian middle-aged adults; it would be of interest to study a cohort across a broader spectrum of age, ethnicity, and body adiposity. Participants were advised to consume a meal that was in accordance with the UK Eatwell Guide the evening prior to the study day; however, we cannot exclude the possibility that differences in habitual macronutrient intake may have influenced our fasting measures of DNL and GNG. It has previously been demonstrated in 6 healthy males that consumption of a high compared to low carbohydrate diet resulted in higher postabsorptive rates of glycogenolysis without any change in the rate of gluconeogenesis.^52^ Therefore, we cannot exclude the possibility that some participants were consuming a high carbohydrate diet, and this may have contributed to the higher glycogenolysis we observed between the respective groups. It would be of interest to explore the effect of dietary composition on the rates of glycogenolysis and gluconeogenesis further. It is also plausible that classifying participants based on a single cutoff led to a modest separation of the two groups and limited the power to detect differences in DNL and GNG which may have been seen when comparing groups with more extreme phenotypic differences.

While our values for frGNG tended to be lower than what has previously been reported,^53-55^ internal validation of our method with that of Chacko et al.^23^ indicated that this was not due to the use of a different glucose derivative as a similar frGNG was observed using their derivative. Instead, differences may be due to the length of the overnight fast being shorter at 10-12 hours, whereas most other studies have reported an overnight fast between 14 and 16 hours.^53,55^ The shorter fasting period and single point measure in our study may not provide a true reflection of fasting frGNG, and it would be of interest to assess frGNG over a longer fasting period with multiple timepoints. In our study, female participants had a significantly lower frGNG than the male participants, which may, in part, explain the lower levels of frGNG in our cohort, which was predominantly female compared to previous studies, which have been predominantly male.^54,55^ Measuring frGNG using ^2^H_2_O may not account for the exchange of carbons via transaldolase or the release of newly synthesized glucose that is stored as glycogen before its release,^56^ although previous studies have found frGNG estimates to be comparable to those using ^13^C-NMR, which would not be influenced by these processes,^57^ and it is likely that these effects would be consistent across the entire cohort.^58^

Taken together, we did not observe differences in the proposed metabolic markers of selective hepatic IR in the fasting state in a cohort of UK adults, across a range of adiposity, when defined by liver fat content and plasma insulin concentrations. However, there was evidence that one of the pathways may be associated with whole-body IR, as in those with HI, hepatic frDNL was higher compared to NI. Although individuals with HI and HF did appear to have higher hepatic glucose production than NI and LF, we did not find this to be exclusively from GNG, and therefore glucose production from GLY from both hepatic and other tissues should be considered.

## Supplementary Material

lvae123_Supplementary_Data

## Data Availability

All data relevant to this study are included in the article or uploaded as supplementary information.
