# Supplementary material for: Dissociation between liver fat content and fasting metabolic markers of selective hepatic insulin resistance in humans
Source: Eur J Endocrinol. 2024 Oct 1;191(4):463–72. doi: 10.1093/ejendo/lvae123 (PMC11497584; doi:10.1093/ejendo/lvae123)
Supplement: lvae123_Supplementary_Data [file lvae123_supplementary_data.pdf]

Supplemental material for review

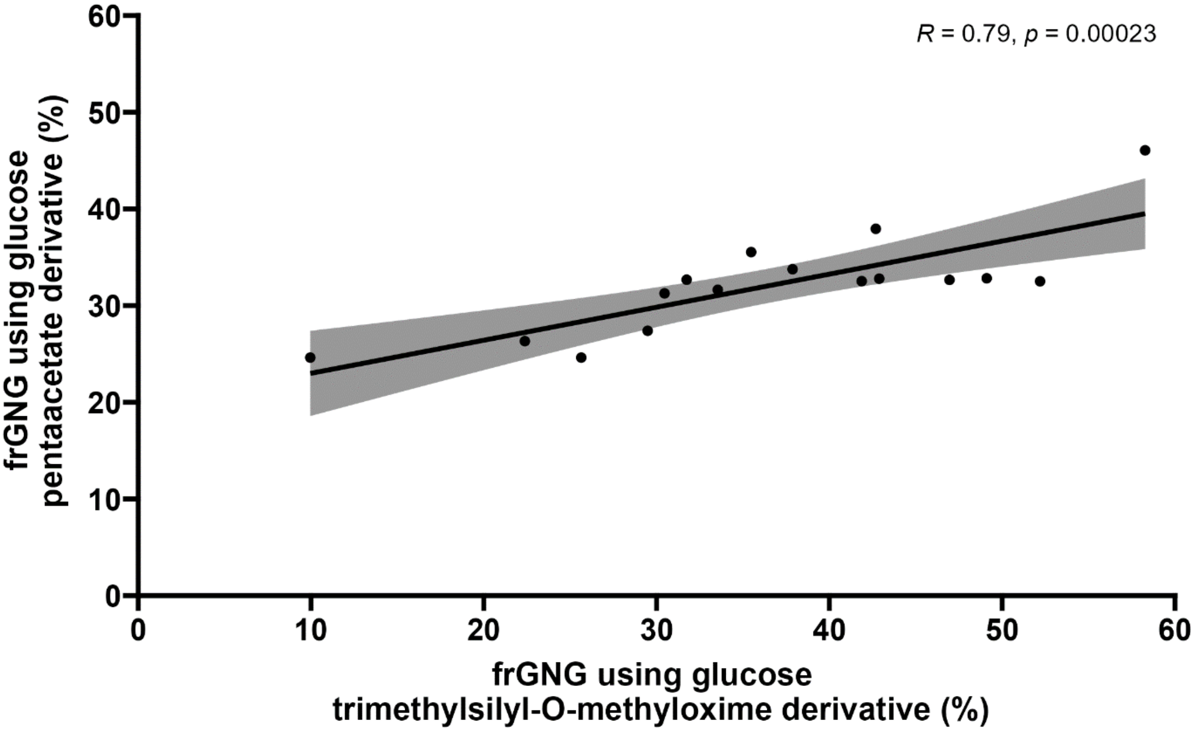

Supplementary figure S1. Validating the use of the glucose trimethylsilyl-O-methyloxime derivative to calculate fractional gluconeogenesis. To validate the calculation of fractional gluconeogenesis (frGNG) using our derivative (trimethylsilyl-O-methyloxime) compared to that of Chacko et al.[23], plasma samples were run using both protocols and the results were correlated.

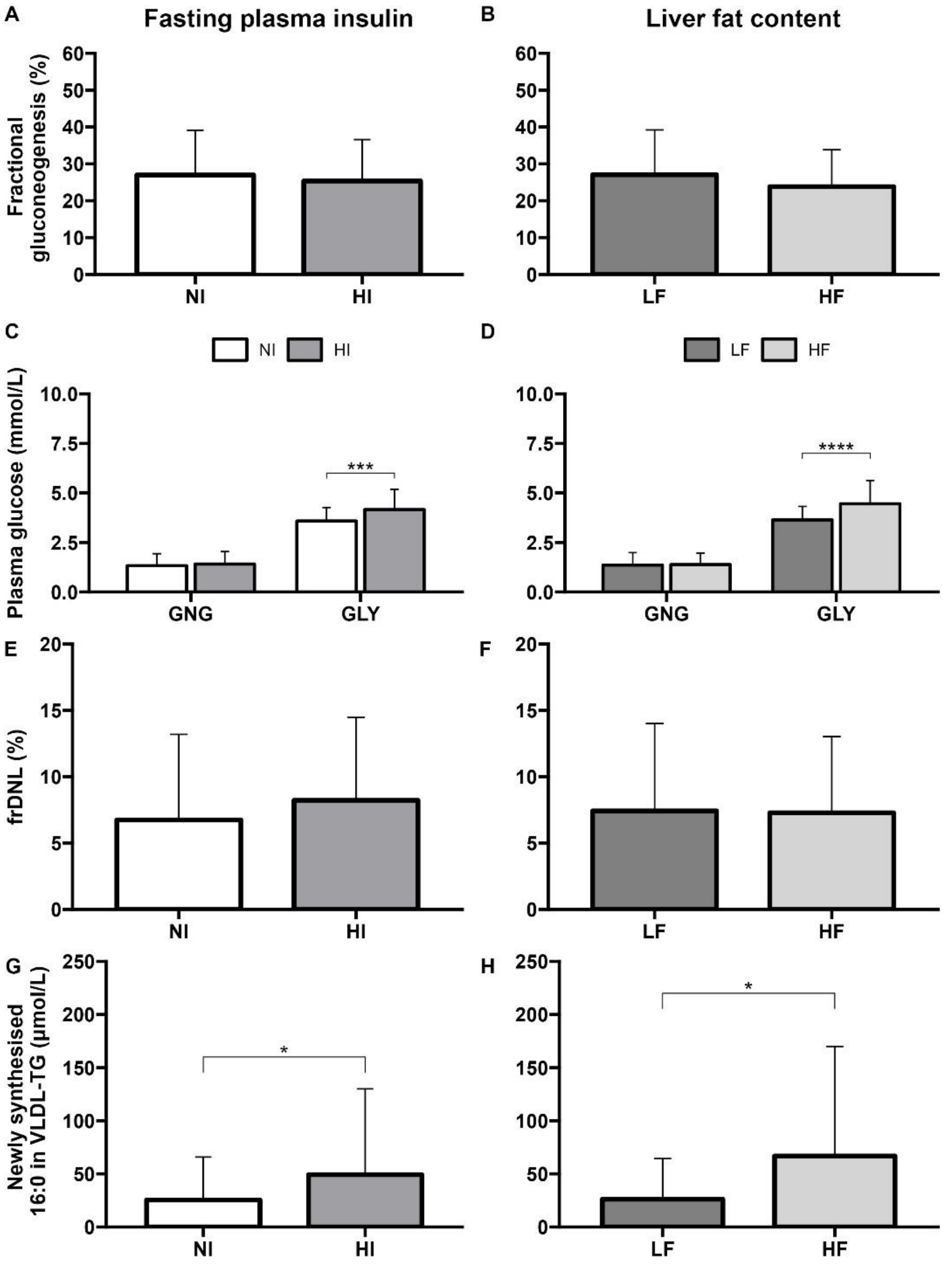

Supplementary figure S2. The effect of fasting plasma insulin and liver fat content on fasting GNG, GLY and DNL in female participants. Female participants were classified as normoinsulinemic or hyperinsulinemic (NI or HI) and as having low or high liver fat content (LF or HF). (a-b) Fractional gluconeogenesis (frGNG) was measured across the groups and (c-d) the absolute contributions of gluconeogenesis (GNG) and glycogenolysis (GLY) to fasting plasma glucose concentration were calculated. (e-f) Fractional *de novo* lipogenesis across groups (frDNL) and (g-h) absolute contributions of newly synthesized 16:0 to very-low-density lipoprotein-triglyceride (VLDL-TG). $^{*}p<0.05$, $^{**}p<0.01$, $^{***}p<0.001$, $^{****}p<0.0001$ or *p*-value stated.

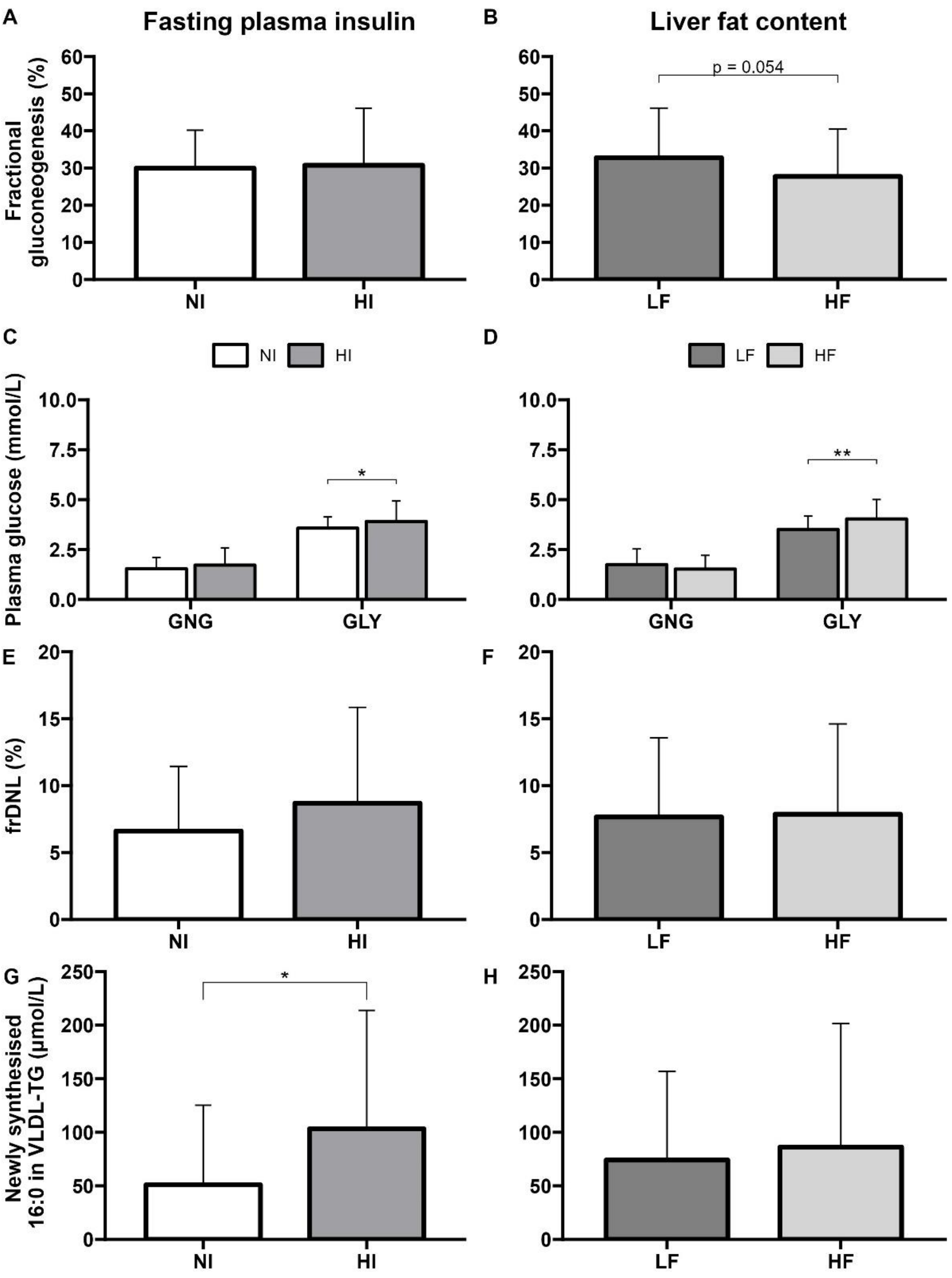

Supplementary figure S3. The effect of fasting plasma insulin and liver fat content on fasting GNG, GLY and DNL in male participants. Male participants were classified as normoinsulinemic or hyperinsulinemic (NI or HI) and as having low or high liver fat content (LF or HF). (a-b) Fractional gluconeogenesis (frGNG) was measured across the groups and (c-d) the absolute contributions of gluconeogenesis (GNG) and glycogenolysis (GLY) to fasting plasma glucose concentration were calculated. (e-f) Fractional *de novo* lipogenesis across groups (frDNL) and (g-h) absolute contributions of newly synthesized 16:0 to very-low-density lipoprotein-triglyceride (VLDL-TG). $^{*}p<0.05$, $^{**}p<0.01$, $^{***}p<0.001$, $^{****}p<0.0001$ or *p*-value stated.

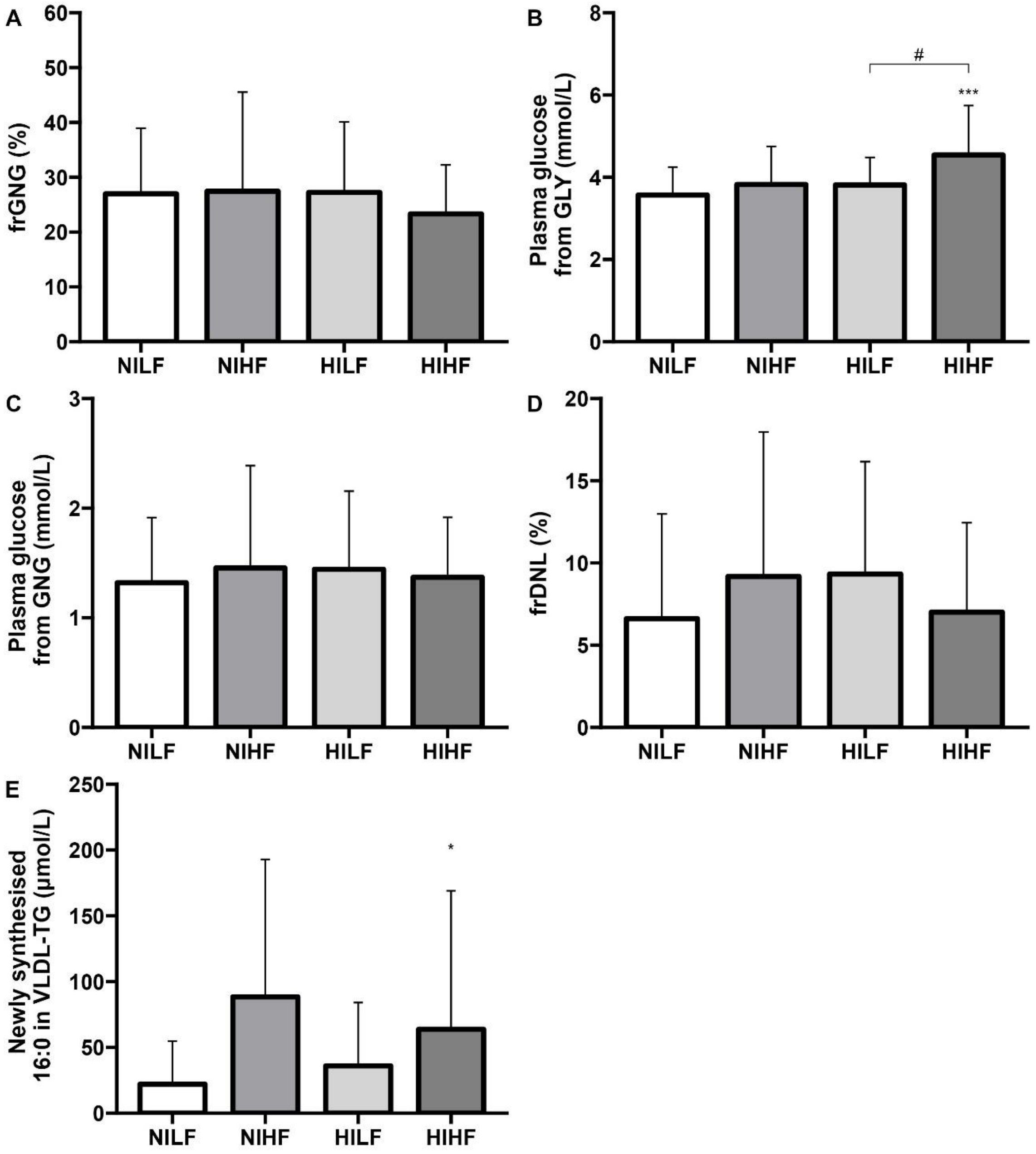

Supplementary figure S4. Further phenotypic grouping in female participants. Female participants were grouped as normoinsulinemic with low liver fat content (NILF), normoinsulinemic with high liver fat content (NIHF), hyperinsulinemic with low liver fat content (HILF) or hyperinsulinemic with high liver fat content (HIHF). (a) Fractional gluconeogenesis (frGNG) was measured and the absolute contributions of (b) glycogenolysis (GLY) and (c) gluconeogenesis (GNG) to fasting plasma glucose concentration were calculated. (d) Fractional *de novo* lipogenesis across groups (frDNL) and (e) absolute contributions of newly synthesized 16:0 to very-low-density lipoprotein-triglyceride (VLDL-TG). $^{*}p$<0.05, $^{**}p$<0.01, $^{***}p$<0.001, $^{****}p$<0.0001 compared to either NILF or LGLD group and $^{\#\#}p$<0.01, $^{\#\#\#}p$<0.001, $^{\#\#\#\#}p$<0.0001 for all other comparisons shown or *p*-value stated.

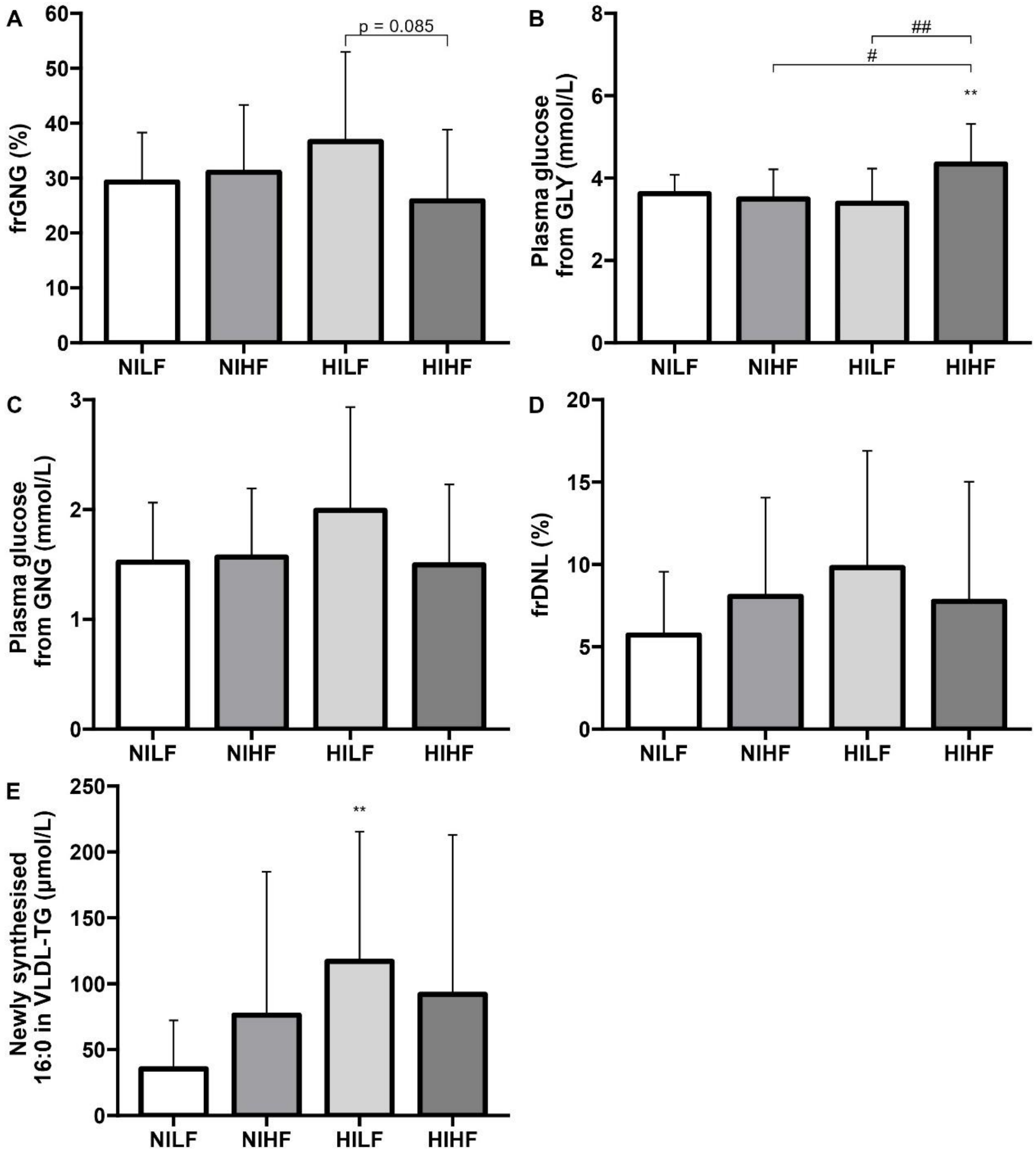

Supplementary figure S5. Further phenotypic grouping in male participants. Male participants were grouped as normoinsulinemic with low liver fat content (NILF), normoinsulinemic with high liver fat content (NIHF), hyperinsulinemic with low liver fat content (HILF) or hyperinsulinemic with high liver fat content (HIHF). (a) Fractional gluconeogenesis (frGNG) was measured and the absolute contributions of (b) glycogenolysis (GLY) and (c) gluconeogenesis (GNG) to fasting plasma glucose concentration were calculated. (d) Fractional *de novo* lipogenesis across groups (frDNL) and (e) absolute contributions of newly synthesized 16:0 to very-low-density lipoprotein-triglyceride (VLDL-TG). $^{*}p<0.05$, $^{**}p<0.01$, $^{***}p<0.001$, $^{****}p<0.0001$ compared to either NILF or LGLD group and $^{\#\#}p<0.01$, $^{\#\#\#}p<0.001$, $^{\#\#\#\#}p<0.0001$ for all other comparisons shown or *p*-value stated.
